# Supplementary material for: Multi‐dataset identification of innovative feature genes and molecular mechanisms in keratoconus
Source: J Cell Mol Med. 2024 Sep 19;28(18):e70079. doi: 10.1111/jcmm.70079 (PMC11412914; doi:10.1111/jcmm.70079)
Supplement: Supplementary file 2 — Material S2. [file JCMM-28-e70079-s002.zip › 7.GSEA analysis/5.RNF217-AS1/KEGG_GLYCOSPHINGOLIPID_BIOSYNTHESIS_LACTO_AND_NEOLACTO_SERIES.html]

Details for gene set KEGG\_GLYCOSPHINGOLIPID\_BIOSYNTHESIS\_LACTO\_AND\_NEOLACTO\_SERIES[GSEA]

|  || Dataset | RNF217-AS1.group.cls#h\_versus\_l |
| Phenotype | group.cls#h\_versus\_l |
| Upregulated in class | l |
| GeneSet | KEGG\_GLYCOSPHINGOLIPID\_BIOSYNTHESIS\_LACTO\_AND\_NEOLACTO\_SERIES |
| Enrichment Score (ES) | -0.56298727 |
| Normalized Enrichment Score (NES) | -1.6438969 |
| Nominal p-value | 0.013972056 |
| FDR q-value | 0.3966524 |
| FWER p-Value | 0.527 |
Table: GSEA Results Summary

  

Fig 1: Enrichment plot: KEGG\_GLYCOSPHINGOLIPID\_BIOSYNTHESIS\_LACTO\_AND\_NEOLACTO\_SERIES      
 Profile of the Running ES Score & Positions of GeneSet Members on the Rank Ordered List

  

| SYMBOL | TITLE | RANK IN GENE LIST | RANK METRIC SCORE | RUNNING ES | CORE ENRICHMENT || 1 | B3GALT2 | na | 4273 | 0.242 | -0.0690 | No |
| 2 | ABO | na | 7486 | 0.140 | -0.1302 | No |
| 3 | B3GALT5 | na | 8179 | 0.124 | -0.1225 | No |
| 4 | ST8SIA1 | na | 9265 | 0.100 | -0.1315 | No |
| 5 | FUT7 | na | 10104 | 0.085 | -0.1368 | No |
| 6 | B4GALT1 | na | 10311 | 0.081 | -0.1246 | No |
| 7 | B3GNT3 | na | 13827 | 0.032 | -0.2186 | No |
| 8 | B4GALT2 | na | 15023 | 0.019 | -0.2489 | No |
| 9 | FUT3 | na | 16166 | 0.007 | -0.2802 | No |
| 10 | B3GNT5 | na | 17723 | -0.008 | -0.3232 | No |
| 11 | FUT6 | na | 18076 | -0.011 | -0.3307 | No |
| 12 | B4GALT4 | na | 18359 | -0.014 | -0.3357 | No |
| 13 | ST3GAL4 | na | 20489 | -0.035 | -0.3893 | No |
| 14 | FUT1 | na | 21565 | -0.046 | -0.4100 | No |
| 15 | FUT2 | na | 22357 | -0.055 | -0.4207 | No |
| 16 | B4GALT3 | na | 22689 | -0.058 | -0.4172 | No |
| 17 | B3GNT2 | na | 23450 | -0.067 | -0.4240 | No |
| 18 | FUT9 | na | 23473 | -0.068 | -0.4095 | No |
| 19 | ST3GAL6 | na | 28748 | -0.169 | -0.5236 | Yes |
| 20 | B4GAT1 | na | 30116 | -0.210 | -0.5160 | Yes |
| 21 | FUT4 | na | 30418 | -0.220 | -0.4754 | Yes |
| 22 | FUT5 | na | 30680 | -0.229 | -0.4317 | Yes |
| 23 | ST3GAL3 | na | 31253 | -0.252 | -0.3919 | Yes |
| 24 | GCNT2 | na | 32311 | -0.310 | -0.3531 | Yes |
| 25 | B3GNT4 | na | 34308 | -0.560 | -0.2854 | Yes |
| 26 | B3GALT1 | na | 34725 | -1.331 | 0.0001 | Yes |
Table: GSEA details [plain text format]

  

Fig 2: KEGG\_GLYCOSPHINGOLIPID\_BIOSYNTHESIS\_LACTO\_AND\_NEOLACTO\_SERIES      
 Blue-Pink O' Gram in the Space of the Analyzed GeneSet

  

Fig 3: KEGG\_GLYCOSPHINGOLIPID\_BIOSYNTHESIS\_LACTO\_AND\_NEOLACTO\_SERIES: Random ES distribution      
 Gene set null distribution of ES for **KEGG\_GLYCOSPHINGOLIPID\_BIOSYNTHESIS\_LACTO\_AND\_NEOLACTO\_SERIES**
